# Regulatory T Cells in Autoimmunity and Cancer: A Duplicitous Lifestyle

**DOI:** 10.3389/fimmu.2021.731947

**Published:** 2021-09-03

**Authors:** Aikaterini Hatzioannou, Athina Boumpas, Miranta Papadopoulou, Iosif Papafragkos, Athina Varveri, Themis Alissafi, Panayotis Verginis

**Affiliations:** ^1^Institute for Clinical Chemistry and Laboratory Medicine, Faculty of Medicine, Technische Universität Dresden, Dresden, Germany; ^2^Center of Clinical, Experimental Surgery & Translational Research, Biomedical Research Foundation of the Academy of Athens, Athens, Greece; ^3^Institute of Molecular Biology and Biotechnology (IMBB), Foundation for Research and Technology - Hellas (FORTH), Heraklion, Greece; ^4^Laboratory of Immune Regulation and Tolerance, Division of Basic Sciences, Medical School, University of Crete, Heraklion, Greece

**Keywords:** regulatory T cell, autoimmune disease, cancer, tolerance, immunotherapy

## Abstract

Regulatory T (Treg) cells, possess a strategic role in the maintenance of immune homeostasis, and their function has been closely linked to development of diverse pathologies including autoimmunity and cancer. Comprehensive studies in various disease contexts revealed an increased plasticity as a characteristic of Treg cells. Although Treg cell plasticity comes in various flavors, the major categories enclose the loss of Foxp3 expression, which is the master regulator of Treg cell lineage, giving rise to “ex-Treg” cells and the “fragile” Treg cells in which *FOXP3* expression is retained but accompanied by the engagement of an inflammatory program and attenuation of the suppressive activity. Treg cell plasticity possess a tremendous therapeutic potential either by inducing Treg cell de-stabilization to promote anti-tumor immunity, or re-enforcing Treg cell stability to attenuate chronic inflammation. Herein, we review the literature on the Treg cell plasticity with lessons learned in autoimmunity and cancer and discuss challenges and open questions with potential therapeutic implications.

## Introduction

Over the last decades T regulatory (Treg) cells have emerged as a novel regulator of the immune system and several approaches have been proposed for their therapeutic targeting in autoimmune diseases, transplantation and cancer. For example, daily administration of low doses interleukin 2 (IL-2) has been linked to expansion of Treg cells and amelioration of graft-vs-host disease as well as induction of remission in systemic lupus erythematosus (SLE) and type I diabetes. On the other hand, immune checkpoint immunotherapy (ICI) in cancer is based on targeting molecules that are abundantly expressed by Treg cells, such as cytotoxic T-lymphocyte-associated protein 4 (CTLA-4) and program cell death protein 1 (PD-1), suggesting that therapeutic efficacy may depend on this powerful suppressive cell subset. Accumulating knowledge however, points to an increased plasticity of the Treg cell compartment expressed with multiple “faces” including loss of suppressive function, expression of inflammatory cytokines and re-programming of their transcription program. Although it remains unclear which factors dictate Treg cell plasticity it is possible that specific microenvironments imprint on Treg cell fate. Therefore, understanding the mechanisms that mediate Treg cell plasticity is of paramount importance and should be considered during the design of Treg cell therapeutic protocols as well as other treatments that directly or indirectly influence Treg cell homeostasis. In this review we discuss current knowledge on Treg cell plasticity with emphasis in autoimmunity and cancer.

## Treg Cell Identity Card

Treg cells constitute the immunosuppressive subpopulation of CD4^+^ T cells, representing approximately 5-10% of peripheral CD4^+^ T cells in blood of healthy individuals (1, 2). They are characterized by the expression of the transcription factor forkhead P3 (FOXP3) (3, 4), a transcription factor instrumental for the development and function of these cells. To this end, individuals, and specifically men, bearing loss-of function mutation in their *FOXP3* gene, have been reported to develop severe systemic multi-organ inflammation and autoimmune disorder, known as immune dysregulation, polyendocrinopathy, enteropathy, X-linked (IPEX) syndrome (5, 6). Similar to human, murine hemizygous males with an X-linked frame shift mutation in their *Foxp3* gene manifest a scurfy phenotype, characterized by hyperactivation and expansion of autoreactive CD4^+^ T cells leading to a lethal inflammatory multi-organ failure (3, 7, 8). In accordance, ectopic expression of Foxp3 confers suppressor function on T effector cells proving the importance of this transcription factor as a critical regulator of Treg development and function (3). Specifically, Foxp3 binds to many genes and acts as both a transcriptional activator and repressor regulating the expression of genes encoding nuclear factors that control gene expression and chromatin remodeling (9). The capacity of Foxp3 to both activate and repress transcription is content and partner-dependent. Thus, it acts as an activator when complexed with the transcriptional factors RELA, IKZF2 and KAT5 and as a repressor when complexed with histone methyltransferase EZH2 and transcription factors YY1 and IKZF3 (10). Moreover, Foxp3 facilitates the formation of repressive chromatin in Treg cells upon their activation in response to inflammatory cues (11). For instance Foxp3 represses cyclic nucleotide phosphodiesterase 3B, affecting genes responsible for Treg cell homeostasis and amplifies molecular features of Treg cells, such as anergy and dependence on paracrine IL-2 (12). On the other hand other studies have demonstrated that Foxp3 defines Treg cell identity indirectly by fine-tuning the activity of other major chromatin remodeling TFs such as TCF1 (13). Up to date two subsets of Foxp3-expressing Tregs have been described: those emerging *de novo* in the thymus (“thymic” or tTregs) and those induced in the periphery (“peripheral” or pTregs).

Apart from *FOXP3* expression, Treg cells abundantly express CD25 (IL-2Rα), which is the low-avidity IL-2 receptor and is crucial for the development and the maintenance of Treg cells (14–16). They also express co-inhibitory molecules such as PD-1, CTLA-4, T cell immunoreceptor with Ig and ITIM domains (TIGIT), V-domain Ig suppressor of T cell activation (VISTA), T cell immunoglobulin mucin 3 (TIM-3) and lymphocyte activation gene-3 (LAG-3) as well as co-stimulatory molecules, such as glucocorticoid-induced TNFR-related protein (GITR), 4-1BB (CD137), inducible T cell co-stimulator (ICOS) and OX-40 (CD134). These molecules are responsible for Treg cell suppressive function and/or activation while their manipulation has been closely linked to Treg cell functional instability in diverse disease settings (17).

Multiple mechanisms have been described *via* which Tregs exert their suppressive activity and can be broadly classified into four distinct categories: 1) secretion of immunosuppressive cytokines, 2) cytolysis, 3) metabolic disruption, 4) suppression of dendritic cells (DC) maturation and function. In more details:

### Immunosuppressive Cytokine Secretion

Inhibitory cytokines, including IL-10, tumor growth factor β (TGF-β), IL-35, are abundantly secreted by Treg cells, orchestrating their function. For instance, IL-10 production by Treg cells decreases the interferon (IFN)-γ-dependent activation of antigen presenting cells (APCs), suppresses IFN-γ production in CD8^+^ cells and induces downregulation of major histocompatibility complex (MHC) II and CD86 in tumor-associated macrophages (18, 19). Similarly, IL-10-producing Treg cells control autoimmunity (20, 21). TGF-β secretion by Treg cells exerts a plethora of immunosuppressive effects, including blockade of DC priming and lymphocyte survival, favoring an anti-inflammatory phenotype in macrophages and inhibiting natural killer (NK) cell effector function in the context of both autoimmunity and cancer (22–24). Lastly, IL-35 secretion has been described to induce cell cycle arrest in T cells through the janus kinases (JAK) - signal transducer and activator of transcription proteins (STAT) pathway, thus potentiating inhibition of T cell proliferation in the tumor microenvironment (TME) and suppression of autoimmune diseases, such as colitis (25).

### Cytolysis

Cytolysis is a Treg cell suppressive mechanism described mainly in cancer and *in vitro* studies. Targeted cells (CD4^+^, CD8^+^ effector T cells, B cells, and NK cells) are driven to apoptosis by Treg cell secreted granzymes in either perforin-dependent or independent manner. Mechanistically, activated Treg cells, through the expression of tumor necrosis factor-related apoptosis-inducing ligand (TRAIL), bind to death receptor (1) on target cells leading to apoptotic-mediated cytolysis (26–28). This is the mechanism exploited by tumor-infiltrating Treg cells to trigger apoptosis in NK cells (29), B cells, DC and cytotoxic T cells of the TME (26, 30, 31).

### “Metabolic Disruption”

Treg cells have the ability to modulate effector cell function by interfering with cell metabolism in an antigen-non-specific manner. To this end, IL2R-expressing Treg cells consume the surrounding IL-2, negatively affecting CD4+ and CD8+ cell proliferative response (32). In cancer, Treg cells express high levels of CD25, actively consuming IL-2, suppressing the activation and proliferation of effector T cells (33) and promoting their apoptosis (34). Induction of apoptosis in autoreactive T cells due to Treg cell-induced IL-2 deprivation has also been described in the T cell adoptive transfer model of inflammatory bowel disease (35). Another well-described suppressive Treg cell mechanism is the production of adenosine from the conversion of extracellular adenosine triphosphate (ATP) by the ectonucleotidases CD39 and CD73 expressed on the cell surface of Treg cells. CD39 expression is driven by Foxp3 and its catalytic activity is strongly enhanced by T-cell receptor (TCR) ligation (36). Interestingly, in the TME, apoptotic Treg cells are the source of extracellular ATP, which is subsequently metabolized into adenosine by live Treg cells (37). Adenosine is a metabolite, which suppresses T cell, DC and pro-inflammatory macrophage maturation and function (17, 33, 38, 39). In support, patients with the remitting/relapsing form of multiple sclerosis (5) have strikingly reduced numbers of CD39^+^ Treg cells in the blood (40).

### Suppression of DC Maturation and Function

A major mechanism of Treg cell-mediated immunosuppression is the inhibition of the immunological synapse between effector T cells and APCs, resulting in impaired APC maturation and T cell anergy. Treg cells, through expression of inhibitory receptors (e.g., CTLA-4), engage the co-stimulatory molecules CD80/CD86 on DC with a higher affinity than CD28, impeding DC maturation and function (41, 42). Furthermore, through CTLA-4, Treg cells capture co-stimulatory molecules on DC by the process of transendocytosis (43), while *in vitro* assays have shown that Tregs down-regulate CD80 and CD86 expression in DC in a CTLA-4 and lymphocyte function-associated antigen (LFA)-1 dependent manner, highly blocking or weakening the signaling between APCs and anti-tumor specific T cells (44). Additionally, Treg-CTLA-4 increases Indoleamine-pyrrole 2,3-dioxygenase (IDO) expression in the DC, which lowers the concentration of tryptophan necessary for T effector cells to proliferate (45). In accordance, our group recently demonstrated that Foxp3^+^ Treg cells potently suppress autoimmune responses *in vivo* through inhibition of the autophagic machinery in DC in a CTLA-4-dependent manner (46). Moreover, Treg cells have been demonstrated to accomplish prolonged interactions with DC in an neuropilin (Nrp)-1/MHC II dependent fashion, a process able to further limit the access of effector T cells (47). Finally, through expression of LAG-3, which is a homolog for CD4, Treg cells have been reported to suppress DC function by LAG-3/MHC II interactions (48).

## Treg Cell Metabolism

Cellular metabolism has emerged as a crucial parameter to influence Treg cell lineage stability, survival, proliferation and function in immune homeostasis but also during pathological situations (23, 33, 45, 49–52). Treg cells exhibit a unique metabolic signature compared with conventional effector T cells. Specifically, meeting of the energy needs of Treg cells suppressive activity, is based on mitochondria metabolism and mainly on elevated levels of fatty acid oxidation (32, 53, 54). As an example, Treg cells are characterized by a metabolic advantage in the nutrient-deprived, lactate-rich, highly hypoxic TME, compared to CD4^+^ T effector and CD8^+^ cytotoxic T cells, which rely primarily on anabolism and glycolysis to support their bioenergetic needs (23). Transcriptomic analysis has shown that human intra-tumoral Treg cells upregulate genes related to lipid synthesis, while in tumor mouse models Treg cells display increased fatty acid synthesis (55); in fact, Foxp3 expression in T cells has been shown to induce oxidative phosphorylation and suppress glycolysis in mouse models (33). Overall, metabolic signaling has emerged as a main component in defining Treg cell function and fate. Thus, understanding how the microenvironment affects the metabolic decisions of Treg cells may help in the delineation of pathogenic mechanisms and can pave the way for novel immunotherapeutic approaches.

## Genetic and Epigenetic Program of the *Foxp3* Locus

### Regulatory Elements of the *Foxp3* Locus

The human *FOXP3* gene is located in the p-arm of the X chromosome and is one of the most intensively studied genes in recent years. The *FOXP3* promoter, positioned in the 1st intron, relies on other cis-regulatory elements. Comparative genomic approaches discovered four conserved non-coding sequences (CNSs) on *Foxp3* locus: 1) regulatory CSN0, located on an intron of the neighbouring gene 5′ of the *Foxp3* locus, 2) intronic enhancer CNS1, located in the 1st intron, along with, 3) CNS2, known as Treg cell-specific demethylated region, 4) CNS3, located directly after exon 1 (56). CNS0 is the most recently discovered regulatory element/super-enhancer, contributing to tTReg cells generation; also regulated by CNS3 (51, 53, 57). On the other hand, CNS1 is redundant for nTreg cell development, while also related to the development of iTreg cells. CNS2 contains highly conserved CpG motifs, known as Treg cell specific demethylated regions (TSDR) that represent the most definitive marker of commitment to the Treg cell lineage (58–61). Importantly, it has been demonstrated that CNS2 deletion affects the stability of *Foxp3* expression during proliferation (56, 57, 62–65).

### Transcription Factors Binding to *FOXP3* Regulatory Elements

Several transcription factors have been described to bind either to the *FOXP3* promoter or to the CNS regions to induce or maintain *FOXP3* expression. The FOXO family of transcription factors directly binds to CNS1, CNS3 areas and indirectly regulates Treg cell-specific genes, *SMAD3* and nuclear factor of activated T-cells (*NFAT*) (66, 67). NFAT binds to CNS1 upon TCR-signalling, while SMAD3 after TGF-β binding (65). Furthermore, Stat5, which is activated upon IL-2 signalling, binds to CNS2, protecting Treg cell identity from other cytokine signals and maintaining heritable transcription of *Foxp3* (68).

### Epigenetic Regulation

Post-translational mechanisms regulate *Foxp3* expression positively and negatively through methylation, acetylation, phosphorylation, and ubiquitination. Transcription factors cAMP response element-binding protein (CREB)/activating transcription factor (ATF), nuclear factor ‘kappa-light-chain-enhancer’ of activated B-cells (NF-κB), Ets-1 and the Runx-Foxp3 complex, all Foxp3-inducers, cannot bind to CNS2 without demethylation (56, 59, 69). Environmental factors, such as Vitamin C, induces CNS2 demethylation in Treg cells in a ten-eleven-translocation 2 (Tet-2)-dependent manner (70). Additionally, TSDR demethylation is facilitated by superagonist CD28, high expression of CD45RA or CD39, and IL-2/CD25 (10, 71, 72). Histone modification also contributes to *Foxp3* expression, with trimethylation of H3K4 on the promoter and CNS1 regions being strongly correlated with *Foxp3* expression in fully differentiated Treg cells (73, 74). Opposingly, TDSR methylation destabilizes *Foxp3* expression and impairs Treg cells suppressive activity. Acetylation is also associated with Treg cells stability and function either by Foxp3 or histone acetylation. TGF-surface signalling assists Foxp3 acetylation (75, 76), while methyl-CpG binding protein two and galectin-9/CD44 pathway promote respectively CNS2-Histone 3 and CNS1-H4 acetylation (77, 78). In contrast, phosphorylation and ubiquitination weaken of their suppressive capacity with respective representative mediators Pim-2 kinase and E3 ligase Stub-1 (79, 80).

## Treg Cell Heterogeneity, Plasticity, and Functional Instability

**Figure 1:**
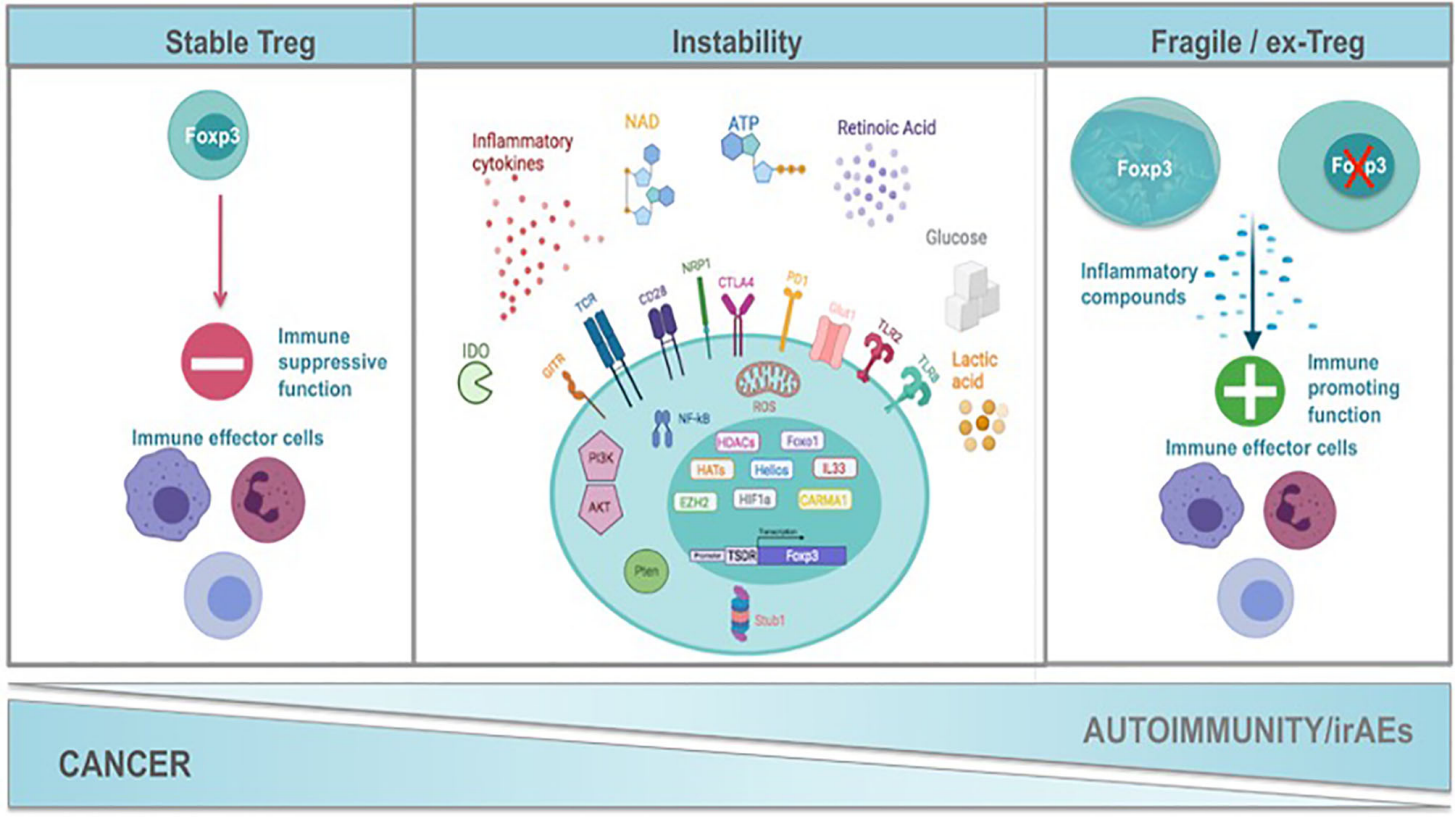
Mechanisms of Treg functional instability constrain cancer while promote autoimmunity and the development of immune related adverse events after immunotherapy. Treg cells exert a strategic role in the maintenance of immune homeostasis. Foxp3 is the hallmark transcription factor of Treg cells orchestrating their function. A complex network of transcription factors such as HELIOS, HIF1a and Foxo1, transcription regulators such as IL33, histone deacetylases, histone methylotransferases, signalosome proteins (CARMA) maintain the transcriptional identity of Tregs and contribute significantly Treg cell stability. Exposure of Tregs to inflammatory stimuli such as inflammatory cytokines (IL1b, IL6, IFNγ, IFNβ, IL12), NAD, ATP and Retinoic Acid results in the alteration of their transcriptional program, in the expression of transcription factors and cytokines that are characteristic of T helper cells and at the end in the attenuation of Treg suppressive function. This may be accompanied by the loss of Foxp3 expression and the generation of ex-Tregs or the generation of fragile Tregs that retain Foxp3 expression. The strength of TCR signaling together with costimulatory and coinhibitory molecules (CD28, GITR, NRP1, CTLA4, PD1) influence downstream pathways such as PI3K/AKT, PTEN, NFkB and the production of ROS by the mitochondria that are critical nominators of the maintenance of Treg transcriptional program and Treg suppressive identity. Oxidative phosphorylation and fatty acid oxidation are metabolic hallmarks of Tregs suppressive function. Any perturbations in the metabolic program of Tregs result in their transformation into non suppressive fragile or ex-Tregs. In autoimmunity the presence of fragile/ex-Tregs burdens the inflammatory response while in cancer stability of Tregs supports the immunosuppressed tumor microenvironment. Immune-checkpoint immunotherapy (ICI) applied in cancer seems to be the destabilization of Tregs and their conversion to fragile or ex-Tregs that may cause the immune-related adverse events (irAEs). Created with BioRender.com.

It is evident that Foxp3, CD25, co-inhibitory and co-stimulatory molecules, immunosuppressive cytokines, death receptors and fatty-acid oxidation define Treg cell identity and suppressive function. Nevertheless, in recent years it is appreciated that Treg cells present a great phenotypic and functional heterogeneity resulting in distinct Treg cell subsets. These subsets seem not to be terminally differentiated since Treg cells may convert from one subset to another under specific stimuli, in accordance to the notion of plasticity that has been described for T helper (78) cells. Specifically, Treg cells can adopt the transcriptional program and functional characteristics of lineage specific T effector cells under inflammatory conditions (81). Multiple subsets of TH-like Tregs have been reported in cancer and autoimmunity settings expressing transcription factors and characteristic cytokines specific for T effector lineage, such as IFNγ^+^Tbet^+^CXCR3^+^ Th1-like Tregs, IL4^+^IL5^+^IL13^+^GATA3^+^ Th2-like Tregs, IL17A^+^RORt^+^ Th17-like Tregs, and CXCR5+Bcl6+ICOS+PD1+ follicular Tregs (TFR) (82–88). However, the function of the Th-like Treg cells remains controversial (81) since it has been shown that some of these Th-like Treg cells lose their suppressive ability, while others become even more suppressive. For instance, Tbet^+^ Treg cells colocalized and inhibited Th1 and CD8 T cell activation and elimination of Tbet-expressing Treg cells resulted in severe Th1 autoimmunity. Conversely, in cancer models Tbet^+^ INFγ^+^ Tregs lose their suppressive function and promote anti-tumor immune responses (89, 90). Th2-like Tregs were the main Treg subset found in tissues and peripheral blood from patients with colorectal cancer and melanoma compared to healthy individuals displaying high viability, activation and suppressive ability conferring to the tumorigenic environment (87). RORγt^+^ Treg cells were derived from Foxp3^+^ thymic Treg cells in an antigen-specific, displayed increased suppressive capacity and efficiently inhibited myelin-specific Th17-cells in a passive experimental autoimmune encephalomyelitis model (91). The main function described for TFR is the suppression of T follicular helper (TFH) cells that support antibody affinity maturation in germinal center reactions and humoral memory formation. Patients with autoimmune rheumatic diseases presented altered numbers of TFR with reduced suppressive function concomitant with a hyperactive phenotype of TFH cells (92). In cancer TFR cells have been found to infiltrate tumors exhibiting superior suppressive capacity and *in vivo* persistence compared to regulatory T cells and their depletion improves tumor control in mice (93). Thus, if the adoption of a Th-like phenotype by Treg cells is a matter of instability or a matter of plasticity remains to be defined. Th-like Treg cells have been characterized as plastic Treg cells in the field of autoimmunity while in cancer they are referred to as fragile Tregs. The common nominator of plastic and fragile Tregs is the production of inflammatory cytokines. In this review we propose the classification of Treg cells into four subpopulations: 1. Treg cells expressing Foxp3, suppressive cytokines and exerting suppressive function, 2. Treg cells expressing Foxp3, producing inflammatory cytokines and retaining their suppressive function, 3. Treg cells expressing Foxp3, producing inflammatory cytokines without exhibiting suppressive function, called from now on fragile Treg cells and 4. the ex-Treg cells that lose Foxp3 expression, produce inflammatory cytokines and do not possess a suppressive function. Both fragile and ex-Treg cells are important players in the pathophysiology of autoimmunity and cancer and will be further reviewed herein (**Figure 1** and **Table 1**).

**Table 1:** Molecules and mechanisms involved in Treg cell stability in cancer and autoimmunity.


Molecules/Procedures responsible for Treg stability	Mechanism	Disease	Type of instable Treg	Reference
CANCER				
**Treg cell lineage-specific molecules**				
FOXP3-TSDR methylation	TET-2 MEDIATED 5mC conversion to 5hmC	colorectal cancer	Ex-Tregs	(94)
histone H3K27 methyltransferase of PRC2	EZH2	Colon adenocarcinoma, melanoma, prostate cancer	Ex-Tregs	(95)
bromodomain-containing proteins	interactions of histone acetyl transferases and (HDACs) with transcription factors and proteins involved in gene expression,	Lung adenocarcinoma	Ex-Tregs	(96)
Helios	zinc-finger transcription factors	Melanoma	Ex-Tregs	(97)
**Inflammatory cytokines**				
IL1b, IL6	Transcription regulator Id2	melanoma	Ex-Tregs	(98)
IFNg		Melanoma	Fragile Th1-like Tregs	(89)
**TCR/CD28 signaling pathway**				
NF-KB		Melanoma	Ex-Tregs	(96)
IL33	NF-KB-TBET	Melanoma	Fragile Th1-like Tregs	(90)
ROS	BACH2 SUMOylation	Colon carcinoma, melanoma	Ex-Tregs	(99)
Mir-126	PI3K/Akt/mTOR	Breast cancer	Ex-tergs	(100)
Nrp-1	Pten/PI3K/Akt	melanoma	Fragile Th1-like Tregs	(89)
PD-1	Pten/PI3K/Akt	Lung tumor	Fragile Th17-like Tregs	(101)
CARMA-1	AP-1, mTOR, NF-κB	Melanoma, colon carcinoma	Fragile Th1-like Tregs	(102)
**Metabolism**				
Lactic acid	MCT-1	Melanoma	Tregs with reduced expression of Nrp-1 and elevated levels of PD1	(103)
Autophagy	Atg5, Atg7	Colon adenocarcinoma	Ex-Tregs	(104)
Glycolysis	Traf3ip3	Colon adenocarcinoma	Ex-Tregs	(105)
TLR8	mTOR/glucose metabolism	Melanoma	Tregs with reduced suppressive function	(106)
IDO	GCN2- kinase dependent production of IL-6 by plasmacytoid DC	Melanoma	Fragile Th17-like Tregs	(107)
**AUTOIMMUNITY**				
**Treg cell lineage-specific molecules**				
CNS2		Multiple autoimmune diseases	Ex-Tregs	(68, 108)
Stub1/USP7/TIP60/Sirtuin1/HDAC7	Proteasomal degradation of Foxp3	Multiple autoimmune diseases	Ex-Tregs	(109–112)
**Inflammatory cytokines**				
IFNβ / IL12 / IFNγ		Multiple sclerosis	Fragile Th1-like Tregs	(113–115)
**TCR/CD28 signaling pathway**				
PI3K/AKT/Foxo1/3		Multiple sclerosis Multiple autoimmune diseases	Fragile Th1-like Tregs	(116, 117)
PTEN/PI3K/AKT	Glycolysis, Foxo, TSDR methylation	Multiple autoimmune diseases, Multiple sclerosis	Th1-like fragile Tregs	(67, 116–118)
ROS	DNA damage response	Experimental autoimmune encephalomyelitis	Dysfunctional Tregs	(119)
**Metabolism**				
Extracellular ATP/NAD+	CD39, CD73		ExTregs	(120, 121)
Glut1		Intenstinal inflammation, lupus	ExTregs	(122, 123)
Ubiquitin ligase E3VHL	Glycolysis/ HIF-1a	Multiple autoimmune diseases, Colitis	Th1-like fragile Tregs	(124)

## Treg Cell Functional Instability in Cancer

Treg cells highly infiltrate tumors, mediating the formation of an immunosuppressive milieu and thus promoting tumor immune evasion (125). The first evidence regarding their function in inhibiting the anti-tumor immunity emerged 20 years ago, when two independent groups demonstrated that elimination of CD25^+^CD4^+^ T cells in mice is associated with enhanced anti-tumor immune responsiveness and tumor regression (126, 127). Several reports demonstrate that an enhanced Treg cell presence in tumor site and peripheral blood of cancer patients was associated with reduced survival and increased metastatic potential in diverse tumor settings (128). On the same line, Treg cell frequencies among tumor infiltrating lymphocytes (TILs) and peripheral blood have been reported to be significantly elevated in nearly all malignancies both in humans and mice, including melanoma, colorectal carcinoma, renal cell carcinoma, pancreatic ductal adenocarcinoma, non-small-cell lung, ovarian epithelial cancer (22, 129–131), gastrointestinal cancer (132), esophageal cancer (133) and breast cancer (134). The accumulation of Treg cells into the tumor niche may involve both the homing of tTreg cells (135), as well as the generation of pTregs (135, 136). The preferential recognition of tumor-specific antigens by the high-affinity TCRs results to clonal expansion, activation and proliferation of Treg cells inside the TME (137).

Many intrinsic and extrinsic factors have been described to induce Treg cell functional instability in the TME. Treg cell lineage specific molecules, TCR/CD28 signaling, metabolism and inflammatory cytokines are factors that have been implicated in the induction of both fragile and ex-Treg cells in cancer resulting in the abolition of the highly immunosuppressive TME and successful control of tumor growth by the immune system. Surprisingly, the same mechanisms that are responsible for the induction of Treg fragility in TME are also those described for the development of ex-Tregs cells (**Figure 1**). The exact mechanisms fine-tuning the decision between the fragile phenotype or the ex-Treg phenotype in the TME are ill defined.

### Treg Cell Lineage-Specific Molecules

One of the aforementioned leading mechanisms safeguarding Foxp3 stability is *FOXP3*-TSDR demethylation, which showed significantly higher rates in tumor sites versus normal sites in patients with colorectal cancer. Increased *FOXP3*-TSDR demethylation in combination with a significant upregulation of STAT5, which is an important transcription factor for regulating *FOXP3* expression, resulted in significantly more *FOXP3* mRNA expression and higher protein synthesis in tumor tissues, serving in the pathogenesis of colorectal cancer. *FOXP3*-TSDR demethylation in tumor-infiltrating CD4^+^ T cells of colorectal cancer patients was mediated by the increase of TET-2 that catalyzed 5-methylcytosine (5mC) conversion to 5-hydroxymethylcytosine (5hmC) (94). Histone modifications are important regulators of chromatin condensation and FOXP3 stability. Pharmacologically or genetically disruption of enhancer of zeste 2 polycomb repressive complex 2 subunit (EZH2) activity, which is a histone H3K27 methyltransferase of the polycomb repressor complex 2 (PRC2) in Treg cells, resulted in the loss of FOXP3 expression and conversion to ex-Tregs cells producing high amounts of pro-inflammatory cytokines, such as TNF-α, IFN-γ, and IL-2 in the tumor tissues but not in lymphoid organs. The lower Treg cell numbers as well as the acquisition of pro-inflammatory functions of tumor-infiltrating FOXP3^+^ Treg cells drove to the remodeling of the TME by enhancing the recruitment and function of CD8^+^ and CD4^+^ effector T cells and protected mice from colon adenocarcinoma (MC-38), melanoma (B16-F10) and prostate cancer (TRAMP-C2) (95). In accordance with Wang D. et al. (95) results on the role of histone modifications in Treg cells stabilization in the TME Xiong Y. et al. showed that prevention of the recognition of histone modifications by the transcriptional machinery ensued ex-Tregs formation, hindering tumor growth in a genetically engineered mouse model of aggressive lung adenocarcinoma (*Kras*^+/LSL-G12D^*Trp53*^L/L^ – KP mice). Specifically, treatment of KP mice with JQ1, a well-characterized inhibitor of the bromodomain-containing proteins that modulates the interactions of histone acetyl transferases (96) and histone deacetylases (HDACs) with transcription factors and proteins involved in gene expression, led to a significant downregulation of Foxp3, CTLA-4, and PD-1 only in lung tumor–infiltrated Treg cells accompanied by decreased suppressive function (138). Nevertheless, JQ1 monotherapy led to minimal or moderate delay in tumor growth, but combination treatment with eitherHDACs inhibitor Ricolinostat (138) or anti-PD-1 immunotherapy (139) significantly delayed tumor growth and improved survival of KP mice. Since JQ1 could also induce functional changes to tumor cells the direct and specific role of bromodomains in Treg cell stability in the TME is still debatable.

Helios, which is a member of the Ikaros family of zinc-finger transcription factors and considered as marker of tTreg cells has been shown to play an essential role in the maintenance of Treg cell program. Accordingly, selective depletion of Helios in Treg cells led to enhanced anti-tumor immunity in the B16F10 melanoma model through induction of an unstable Treg cell phenotype in the TME. Helios-deficient tumor-infiltrating Treg cells produced significant amounts of proinflammatory cytokines (TNF-a, IFN-γ), displayed a nonanergic phenotype, reduced immunosuppressive activity and profoundly restrained Foxp3 and CD25 expression (97).

### Inflammatory Cytokines

Both pro- and anti- tumorigenic effects have been reported for inflammatory cytokines. These contradictory results may be attributed to the divergent role of cytokines on different cells forming the TME as well as to different effects of cytokines depending on tumor stage. Specifically, for Treg cells it has been shown that exposure to inflammatory cytokines such as IL-1β and IL-6 substantially reduced Treg cell stability and enhanced the conversion of Treg cells to ex-Treg Th17 cells *via* the upregulation of the transcription regulator Id2 (98, 140). Treg cell-specific ectopic expression of Id2 (TetRId2EmGFPFoxp3YFP−Cre) resulted in reduced Foxp3^+^ Treg cell infiltration within tumor tissue as well as in tumor-draining lymph nodes, increased the expression of IL-17A within the CD4^+^Foxp3^−^ tumor TILs and arrested tumor growth in B16F10 melanoma-bearing mice (98). These results implied that the presence of inflammatory cytokines in the immunosuppressed TME further potentiates Treg cells stability and function. Interestingly, Overacre-Delgoffe et al. demonstrated that IFN-γ produced by fragile Treg cells could destabilize the suppressive Treg cells infiltrating tumors, a process named by the authors as “infectious fragility”. IFN-γ substantially limited the suppressive capacity of both mouse and human tumor infiltrating Treg cells and not that of peripheral Treg cells (89).

### TCR/CD28 Signaling Pathway

TCR and CD28 stimulation facilitates the activation of Treg cell and is indispensable for the preservation of the activated Treg cell transcriptional signature. Nevertheless, the fine-tuning of the strength of TCR stimulation seems to be pivotal for the maintenance of Treg cells. Treg cells possess a plethora of mechanism to attenuate TCR/CD28 signaling including diminished calcium flux, retained activation of Akt, Foxp3-mediated suppression of *Zap70* transcription and expression of inhibitory receptors such as CTLA-4 and CD5 (141). In the TME TCR/CD28 signaling seems also to denominate Treg stability and promote tumor growth.

NF-κB activation is a key downstream event of TCR/CD28 signaling. Activation of NF-κB occurs through the canonical pathway leading to the activation of NF-κB heterodimers consisting of p50 and p65 or p50 and c-Rel and through the non-canonical pathway leading to nuclear translocation of p52-RelB heterodimers. Genetic ablation or chemical inhibition of c-Rel with an FDA-approved drug, Pentoxfylline (PTXF) but not p65 in melanoma-bearing mice modified the transcriptional landscape of activated Treg cells. Specifically, it caused significantly decreased expression of Treg cell markers such as Foxp3, CD25 and Helios and genes required for optimal Treg cell function and immunosuppression in the TME, such as *Tgfb1* or *Gzmb*. Thus, c-Rel inhibition reduced Treg cell activity in the TME resulting in reinforcement of anti-tumor immunity, attenuation of tumor growth and potentiation of anti-PD-1 therapy without causing autoimmunity (96). Nevertheless, we have recently demonstrated that NF-κB pathway is also responsible for the induction of fragile Treg cells in the TME. In detail, specific genetic depletion of IL-33 in Treg cells, which binds to NF-κB and restricts its transcriptional activity, attenuated Treg suppressive properties *in vivo* and facilitated tumor regression in the B16F10 melanoma model. Absence of IL-33, epigenetically reprogrammed Treg cells to express IFN-γ, consistent with a fragile phenotype, dependent on NF-κB–T-bet axis, while maintaining Foxp3 expression. Importantly, genetic ablation of *Il33* potentiated the therapeutic efficacy of immunotherapy (90).

Several studies have suggested that excessive reactive oxygen species (1, 26) levels are associated with tumor-induced immunosuppression and that ROS can participate in Treg cell-mediated immunosuppression. Likewise, ROS that is induced upon TCR and CD28 activation was found to be increased in tumor-infiltrating Treg cells compared to their splenic counterparts. A recent paper in Nature Communications by Yu X. et al. (99) unraveled the molecular mechanism underlying the cross-talk between ROS and Treg cell-mediated tumor immunosuppression. TCR/CD28 induced ROS led to the accumulation and stabilization of small ubiquitin-related modifier (SUMO)-specific protease 3 (SENP3) in Treg cells repressing T effector cell-specific transcriptional programs and maintaining Treg cell-specific gene signatures by triggering BACH2 deSUMOylation. In detail, genetic deletion of *Senp3* specifically in Treg cells led to the expression of T effector-related genes such as *Ifng*, *Il4*, *Il13*, *Il17a*, *Il22*, and *Il9* and loss of Treg cell-specific genes, such as *Foxp3* and *Pdcd1*. Senp3-induced Treg destabilization resulted in increased frequencies and effector function of CD4^+^ and CD8^+^ T effector cells infiltrating the tumors and reduction of tumor growth in MC38 colon carcinoma model and B16F10 melanoma model. These findings suggested that targeting ROS in Treg cells may be an effective approach to ameliorate tumor immune tolerance (99).

It is well accepted that TCR signaling activates the phosphoinositide 3-kinase (PI3K)/protein kinase B (Akt)/mechanistic target of rapamycin (mTOR) pathway in Treg cells but limited activation of this pathway is crucial for Treg cell suppressive function. Micro-RNAs can regulate these pathways, stabilize Treg cells and thus be potential targets of cancer immunotherapy. For instance, silencing of miR-126 on Treg cells enhanced the expression of its target p85b and subsequently altered the activation of PI3K/Akt pathway leading in reduced expression of Foxp3, CTLA-4, GITR, IL-10 and TGF-b on Treg cells. Mir-126KO Treg cells presented impaired suppressive function and promoted a robust anti-tumor immune response that resulted in a diminished tumor growth in a murine breast cancer model (100). PI3K/Akt/mTOR pathway has been also implicated in the induction of fragile Treg cells in the TME. Specifically, it has been demonstrated that Nrp-1, which is constantly expressed by Treg cells reduced Akt signaling following ligation with semaphorin (Sema)4a and Nrp1-Sema4a interaction promoted Treg cell survival, stability transcriptional program with downregulation of the lineage defining transcription factors Eomes, IRF4 and RORγt. Indeed, specific deletion of Nrp-1 in Treg cells (*Nrp1*^f/f^*Foxp3*^Cre^ mice) or blockade of Nrp-1 with Sema4a mAb, Nrp-1 mAb and Sema4a-Ig significantly decreased tumor growth in the B16F10 melanoma mouse model (29, 89). Nrp1KO Treg cells presented a fragile phenotype characterized by expression of IFN-γ, elevated phospho-Akt, reduced ICOS expression and lack of suppressive activity *in vitro* although retaining Foxp3 expression (89). Mechanistically, Nrp-1 recruited the Phosphatase and tensin homolog (PTEN) to the immunologic synapse, which inhibited PI3K and thus limited phosphorylation of Akt (29). PD-1 which is expressed by tumor activated Treg cells is also an upstream regulator of PTEN restricting Akt activation. The *in vitro* blockade of PD-1 pathway in Treg cells rapidly increased Akt phosphorylation, FoxO3a was lost, and suppression activity was abrogated. Thus, it seems that PTEN played an important role in Treg cell function and stability. Indeed, aggressive melanoma and lung tumors implanted in PTEN-Treg-KO hosts grew much slower accompanied by a robust anti-tumor immunity. The tumor-infiltrating PTENKO Treg cells lost the expression of PD-1 but not Foxp3, expressed proinflammatory cytokines such as IL-2 and IL-17 (101).

Finally, the dominant role of TCR/CD28 and downstream molecules regulation in Treg cell functional stability was further confirmed by Di Pilato et al. who studied how the function of TME Treg cells is altered by CARMA1, which is a scaffold protein of the caspase recruitment domain-containing membrane-associated guanylate kinase protein-1 (CARMA1)–B cell lymphoma (BCL)10–Mucosa-associated lymphoid tissue lymphoma translocation protein 1 (MALT1) (CBM) signalosome complex implicated in activation of AP-1, mTOR, NF-κB and mRNA stabilization in response to TCR. Carma1^-/-^ Treg cells retained expression of Foxp3 but secreted IFN-γ and at lower frequencies IL-4, IL-17 and TNF. Production of IFN-γ by Treg cells induced the activation of the intra-tumoral myeloid cells and increased the antigen presenting capacity of tumor cells, resulting in restrained tumor growth. Importantly, blockade of PD-1 in absence of CARMA1 caused rejection of tumors that otherwise do not respond to anti-PD-1 monotherapy (102).

### Metabolism

Recent studies have revealed that metabolic programs play pivotal roles in controlling Treg cells stability. Treg cells require predominantly fatty-acid oxidation in contrast to effector T cells that are glycolytic. Interestingly, it has recently been demonstrated that inhibition of lipid synthesis in intra-tumoral Tregs of mouse models diminishes tumor progression, enhancing anti-tumor immune responses (142). Thus, in the harsh TME that it is poor of glucose and high in lactic acid, Treg cells possess a survival and functional advantage promoting immunosuppression. Intratumoral Treg cells are adapted to the lactic-enriched TME by the upregulation of CD36 *via* a peroxisome proliferator-activated receptor-β (PPAR-β) signaling (36). Lactic acid was an additional energy source for Treg cells in the TME, up-taken through transporter monocarboxylate transporter (MCT) 4 and converted to pyruvate and NADH (23). In accordance, glycolysis by tumor cells correlated with the suppressive function of intratumoral Treg cells. Sequencing of tumor-draining lymph node and tumor Treg cells that present high glucose avidity from B16F10 melanoma-bearing mice revealed reduced expression of Treg cell signature genes, while retaining Foxp3 expression. Subsequently, tumor-infiltrating Treg cells that are deficient for MCT1, which catalyzes the intake of lactic acid, upregulated glucose consumption. The MCT1KO Treg cells that present a reduced suppressive function *ex vivo*, decreased expression of Nrp-1 and elevated PD-1 levels lost their suppressive function and allowed the control of B16F10 tumor growth. Treg-specific deletion of the lactate transporter resulted in decreased tumor growth and response to ICI (103).

Autophagy and lysosomal function regulate Treg cell metabolic fitness in the TME. Specifically, Treg cell-specific deletion of the autophagy gene *Atg7* or *Atg5* increased glycolytic metabolism, broke Treg cell stability and facilitated tumor clearance (104). On the other hand, Treg cell–specific deletion of lysosomal *Traf3ip3* potentiated mTORC1 signaling, mediated hyper-glycolytic metabolism and impaired Treg cell function. Traf3ip3KO Tregs induced a strong anti-tumor T cell response and a profound reduction in tumor size in the MC38 colon carcinoma model. Interestingly, both *Traf3ip3* and *Atg7/Atg5* deficient Treg cells upregulated the expression of inflammatory cytokines genes, such as *Ifng*, *Il4*, *Il13*, *Il17a*, *Il17f*, and *Il21* and presented impaired transcription of the Treg cell signature gene *Foxp3* (105).

Despite the fact that data on mouse tumor Treg cells strongly suggest the use of lipid oxidation as a primary metabolic pathway the same is not true for human Treg cells. The suppressive function of human tumor-associated Treg cells is predominant dependent on glucose metabolism triggering cell senescence and DNA damage in responder T cells. Disruption of glucose metabolism by toll-like receptor (TLR) 8 signaling in human Treg cells reversed Treg inhibitory functions, enhanced anti-tumor immunity and tumor immunotherapy efficacy in a mouse model of melanoma (106).

The consumption of specific nutrients in the TME is another mechanism for the maintenance of immunosuppression and Treg stability. For instance, IDO (19), an enzyme implicated in tryptophan metabolism, was upregulated in murine plasmacytoid DC in tumor-draining lymph nodes, where it potently activated Treg cells. Pharmacological inhibition of IDO in the B16F10 melanoma model released the GCN2- kinase dependent production of IL-6 by plasmacytoid DC and promoted conversion of Treg cells to the Th17-like phenotype. Th17-like Tregs that expressed IL-17, IL-22, IL-2, TNF and RORγt but in parallel maintained Foxp3 expression markedly enhanced anti-tumor immunity (107).

### Exhaustion

Another important barely studied category of Treg functional instability in the TME is exhaustion. Treg cells in the peripheral blood and tumor of glioblastoma multiform patients, upregulated the PD-1 concomitantly with IFN-γ and molecular signatures of exhaustion. PD-1 Treg cells presented reduced suppression capacity *in vitro* and a partial demethylation at the TSDR locus while they preserved the FoxP3 expression. These data are in contrast to the aforementioned data about the role of PD-1/PD-L1 axis in promoting Treg induction through inhibition of the Akt/mTOR pathway. Nevertheless, human Treg cells presented a different biology compared to murine Treg cells and also the expression of PD-1 in human Treg cells may be induced as a compensatory mechanism to stabilize the PI3K/Akt pathway and repress IFN-γ (143).

## Treg Cell Functional Instability in Autoimmunity

Autoimmune diseases comprise a heterogeneous group of poorly understood long-term disorders that affect approximately 5-8% of the population (144). While each autoimmune disorder is unique, they are all caused by a breakdown of tolerance against endogenous proteins. This leads to auto-inflammatory events that promote the destruction of organs in a humoral and cellular immune mediated manner. Immune suppression by Foxp3^+^ Treg cells is essential and indispensable for maintenance of tolerance and prevention of autoimmunity, as illustrated by spontaneous autoimmune disease development when Treg cells are rendered deficient. Consistently recent studies have highlighted that Treg dysfunction is a common denominator in autoimmunity, with reduced Treg cell frequencies and impaired suppressive function identified in a wide range of autoimmune diseases, including multiple sclerosis (5), SLE, type 1 diabetes, thyroiditis, and inflammatory bowel disease (145–150). Thus, it is becoming apparent that Treg cells possess a unique power in supervising autoimmune reactions and the re-establishment of self-tolerance. Treg cells during autoimmunity may receive ques from the inflammatory environment that imprint on their phenotype and function, leading to acquisition of an unstable phenotype due to either loss of *Foxp3* expression or fragility with maintenance of *Foxp3* expression (**Figure 1**). An in-depth characterization of the mechanisms underlying Treg cell dysfunction in autoimmunity would enable new strategies for managing autoimmune diseases. In this section we will focus on recent literature exploring Treg cell stability and plasticity and their implications for the pathogenesis of autoimmune diseases.

### Ex-Treg Cells in Autoimmunity

Loss of *Foxp3* expression has been shown to contribute to autoimmunity and inflammation in various *in vivo* settings (151–158). Under autoimmune conditions of diabetes, a substantial percentage of cells had unstable expression of *Foxp3* in inflamed tissues. These ‘exFoxp3’ T cells, secreted inflammatory cytokines, acquired an activated-memory phenotype and were able to induce rapid onset of diabetes upon adoptive transfer (155). In a different autoimmune setting, experimental autoimmune encephalitis, immune activation and inflammation driven by self-antigens in the central nervous system, promoted Foxp3 instability exclusively in autoreactive Treg cells during the induction phase of the response, a process that was reversed during the resolution phase of inflammation or upon IL-2-anti-IL-2 complex treatment (158). Furthermore, data from human studies, highlight the importance of the imbalance of Th17/Treg cell ratio as a pathological feature in multiple sclerosis (5), positively correlating with disease severity (159, 160). In this notion, impaired *Foxp3* and *Helios* expression along with increased numbers of CD161^+^Th17 like CD45RA^-^Foxp3^lo^ Treg cells was an early hallmark of multiple sclerosis (161), whereas epigenetic modification of *Foxp3* through histone deacetylase mediated by TLR-2 stimulation induced IL-17 production in Treg cells isolated from multiple sclerosis patients (162). Similarly, Komatsu N. and colleagues demonstrated the pathogenic conversion of Treg cells that lost their *Foxp3* expression into Th17 cells during autoimmune arthritis. Fate mapping analysis showed that IL-17-expressing exFoxp3 T cells accumulated in inflamed joints, expressed Sox4, chemokine (C-C motif) receptor 6 (CCR6), chemokine (C-C motif) ligand 20 (CCL20), IL-23 receptor (IL-23R) and receptor activator of NF-κB ligand (RANKL, also called TNFSF11), in a process mediated by synovial fibroblast-derived IL-6 (153). Among the four CNSs described for the initiation and maintenance of *Foxp3* transcription, CNS2 containing Runx1-CBFβ binding sites, is the only one preventing autoimmunity. In this context, CNS2-deficient mice succumb to development of autoimmunity due to loss of Foxp3 and instability in Treg compartment (68, 108).

Post translational or epigenetic modifications, affect Foxp3 protein expression and thus regulate Treg cell function and development of autoimmunity. Diverse inflammatory stimuli have been shown to promote Lys48-linked ubiquitination mediated by Stub1 ubiquitinase binding to Foxp3, thus targeting it for proteasomal degradation (109). In contrast, under similar inflammatory conditions, USP7 deubiquitinating enzyme expressed in Treg cells, is downregulated resulting in Foxp3 degradation (110), while its conditional deletion in Treg cells leads to lethal autoimmunity (76). Additionally, disruption of the association of other proteins known to mediate Foxp3 acetylation, such as TIP60, Sirtuin 1 or HDAC7, leads to increased polyubiquitination of Foxp3 and development of autoimmune responses (111, 112).

### Fragile Treg Cells in Autoimmunity

Fragility of Treg cells has recently arisen as a hallmark of autoimmune diseases, with Treg cells rendered dysfunctional in their suppressive features whilst expressing pro-inflammatory cytokines, maintaining Foxp3 expression and acquiring Th cell-like phenotypes, with identical transcription factors used by Treg cells to inhibit specific types of immune response (84, 113, 116, 117, 163–165).

Up to date, the best characterized Th-like Treg subset in autoimmunity is the Th1-like Treg cells, with upregulated expression of transcription factor Tbet, chemokines CCR5 and CXCR3, stable Foxp3 expression due to highly demethylated TSDR region and increased production of IFN-*γ* cytokine. Increased frequency of these Th1-like Tregs has been observed in periphery of mouse models and patients with autoimmune diseases, such as type1 diabetes (113, 166), multiple sclerosis (116, 163), autoimmune hepatitis (165) and Sjogren syndrome (167). Following treatment with IFN-β, numbers of IFN-*γ* secreting Th1-like Treg cells are downregulated to physiological levels in individuals with multiple sclerosis (114). Moreover, blocking IFN-*γ* is capable of re-establishing Th1-like Treg cells suppressive function during multiple sclerosis in humans and animal models, whereas neutralization of IL-12 resulted in restraining their generation (113, 115). Mechanistically, using a genome wide gene expression approach Kitz et al. demonstrated that PI3K/AKT/Foxo1/3 pathway was responsible for IFN-γ secretion by human Treg cells. Blockade of this pathway, using multiple sclerosis as their *in vivo model*, inhibited IFN-γ secretion and restored the immune suppressive function of Treg cells (116). In the same path, Ouyang W. and colleagues, demonstrated that mice with depleted Foxo1 expression specifically in Treg cells, developed a fatal auto-inflammatory syndrome without the loss of Foxp3 expression and Treg cells displayed a Th1-like phenotype with loss of *in vivo* suppressive activity (117). Importantly, the same study was able to identify approximately 300 Foxo1-bound target genes, including IFN-γ, that were not directly regulated by Foxp3, implying that separate and autonomous signaling pathways may operate simultaneously driving Treg function in autoimmunity.

The second Th-like Treg subset operating in autoimmune diseases is Th17-like Treg cells. Specifically, identification of increased numbers of IL-17^+^Foxp3^+^ Treg cells in the synovium of individuals with active rheumatoid arthritis (5), suggests that plastic Foxp3^+^ Treg cells contribute to the pathogenesis of rheumatoid arthritis (153). Moreover, IL-17A^+^Foxp3^+^CD4^+^ cells have been observed in skin lesions of patients with severe psoriasis (84) and in experimental models of autoimmunity (168). However, observations concerning Th17-like Treg suppressive function isolated from rheumatoid arthritis patients have been rather contradictory depending on the site of Treg cell isolation. To this end, although high frequencies of IL-17-producing Treg cells were present in the peripheral blood of rheumatoid arthritis patients, these cells were able to suppress T cell proliferation *in vitro*. In contrary, Treg cells isolated from rheumatoid arthritis synovial fluid lost their suppressive function (169).

### Metabolic Cues in Treg Cell Functional Stability During Autoimmunity

Deficiencies in metabolites such as retinoic acid or vitamin D are prevalent in multiple autoimmune syndromes and are established as a risk factor for development of diseases such as multiple sclerosis, rheumatoid arthritis, SLE and type 1 diabetes (170, 171). The same metabolites, however, have been shown to increase stability of Treg cells in diverse experimental settings. To this end retinoic acid can prevent loss of Foxp3 expression during human Treg expansion and in inflammation (172), it directly increases the expression of ERK signaling to promote *Foxp3* expression as well as increases histone methylation and acetylation of the promoter and CNS region of *Foxp3* (173). In a similar manner, Vitamin D metabolites such as 1,25-dihydroxyvitamin D3, promote *FOXP3* expression by binding to newly identified vitamin D response elements in the intronic CNS region of human *FOXP3* gene (174, 175). From the above-mentioned studies a solid hypothesis is that the lack of essential metabolites from vitamins that is characterizing autoimmune diseases can be detrimental to Treg stability and immunosuppressive function.

Other metabolites derived from tryptophan catabolism, initiated by enzyme IDO, are known to promote *Foxp3* expression, through inhibition of IL-6 production by DC (107, 176), whereas altered tryptophan distribution has been identified in a variety of autoimmune settings (177). Moreover, Foxp3 stability can be also regulated by metabolites deriving from extracellular purine metabolism. Thus, during cell damage and inflammation ATP and NAD^+^ molecules are released extracellularly due to enhanced cell lysis and are able to activate P2x7 receptor on Treg surface that in terms limits *Foxp3* expression and induces their conversion to TH17 cells (120). To counterbalance this effect Treg cells are known to express CD39 and CD73 ectonucleotidases on their surface, responsible for converting excess extracellular ATP to adenosine that is immunosuppressive (40). Nevertheless, during autoimmune diseases CD39 and CD73 expression on Treg cells is downregulated possibly providing a link to adenosine and Treg instability in autoimmunity (121).

Cellular metabolism is also closely linked to Treg cell stability and plasticity. As mentioned above Treg cells rely more on mitochondrial metabolism compared to glycolysis to maintain their energy production and suppressive function and *Foxp3* expression orchestrates Treg cell metabolism by suppressing glycolysis and enhancing OXPHOS through mTORC1 (45, 121). In favour of this concept deletion of hypoxia inducible factor (HIF)-1α known to promote glycolysis, leads to increased Foxp3 stability and Treg cell induction (178). In addition, mice overexpressing *Glut1* have reduced *Foxp3* expression during intestinal inflammation (122), while pharmacologic inhibition of Glut1 ameliorates lupus autoimmune phenotype in mice by targeting T cell activation (123). Furthermore, ubiquitin ligase E3VHL deficient Treg cells become IFN-*γ* secreting Th1-like cells through a shift in glycolysis and increased binding of HIF-1α to *Ifng* promoter (124). Bridging Treg cellular metabolic function to autoimmune pathogenicity, our group found a Treg dysfunction recapitulating the features of autoimmune Treg cells, with a prominent mitochondrial ROS signature and importantly, scavenging of Treg mitochondrial ROS production was able to ameliorate experimental encephalomyelitis in mice (119).

Intracellular signalling pathways involved in Treg cell metabolism also play a crucial role in maintaining their stability and controlling their plasticity. PTEN deletion in Treg cells increases PI3K/AKT pathway activation driving enhanced glycolysis, reduced FoxO presence in the nucleus and promoter regions of *Foxp3* and increased methylation of its TSDR region (67, 117, 118). In parallel, enhanced AKT activation in Treg cells has been demonstrated during autoimmune diseases (116, 179, 180), whereas blockade of this pathway in Treg cells isolated from multiple sclerosis patients inhibits IFN-γ secretion and restores the immune suppressive function of Treg cells (116).

## Treg Cell Functional Instability in Cancer Immunotherapy and Autoimmune Related Adverse Events

Despite of the promising results of cancer immunotherapy, its clinical efficacy is limited to the minority of patients, whereas it is usually accompanied by the development of immune related adverse event (irAEs), due to the excessive activation of the immune system, with the underlying mechanisms remaining unknown. Accumulating evidence suggests that the prevalence of Treg cells inside the TME is associated with tumor progression, as well as the development of acquired resistance to cancer immunotherapy and irAEs development (130, 181). Considering the above, recent therapeutic attempts have been focused on the manipulation of Treg cell-mediated immunosuppression in order to enhance anti-tumor immune responses and improve the clinical outcome of cancer patients. Several strategies for targeting tumor associated Treg cells may involve either direct or indirect approaches, that have been tested clinically or/and preclinically, such as: a. the CD25 targeting for Treg cell depletion with either blocking antibodies or a recombinant protein composed of IL-2 and the active domain of the diphtheria toxin (127, 182–187), b. the targeting of Treg-specific co-inhibitory molecules (CTLA-4, PD-1, TIGIT, VISTA, TIM-3, LAG-3) (188–191), with blocking antibodies to specifically deplete or diminish the suppressive function of Treg cells in the TME (143, 192–196), c. The usage of agonists against GITR (197–199), OX-40 (200, 201) and ICOS (202) can drive the attenuation of Treg cell immunosuppressive activity (203), d. targeting of PI3K signaling (204) or molecules like CD39 and CD73- critical regulators of adenosine pathway (205)- which are definitive of Treg cells behavior in the TME, e. inhibition of vascular endothelial growth factor (VEGF)-VEGF receptor 2 (VEGF-VEFGFR2) pathway, which is implicated in the accumulation of Treg cells, reduced their infiltration to the TME (206, 207), f. inhibition of TGF-β pathway, a major mediator of Treg presence in the TME, can diminish the induction of Treg cells (208, 209).

The ultimate goal would be to specifically deplete Treg cells infiltrating tumors without affecting tumor-reactive effector T cells, while suppressing autoimmunity. Getting a better insight into the mechanisms that induce functional destabilization of Treg cells may allow their exploitation as therapeutic tools. Induction of Treg functional instability may prove a more redundant approach in cancer immunotherapy compared to the targeting of one specific Treg suppressive mechanism and with less autoimmune side effects compared to depletion of Treg cells.

There are several pieces of evidence showing that ICI, specifically, anti-PD-1 and anti-CTLA-4 that are currently used in clinical practice may induce a destabilized phenotype in tumor Treg cells. Specifically, peripheral Tregs from patients suffering from glioblastoma multiform presented an exhausted phenotype and increased expression of IFN-γ following treatment with anti-PD-1 (143). Moreover, PD-1 blockade increased IFN-γ production in the TME and as a consequence drove intratumoral Treg fragility (89). Anti-CTLA-4 also induced fragility in intratumoral Treg cells. Anti-CTLA-4 treated Treg cells promoted CD28 co-stimulation leading to decreased Treg cells suppression and increased glucose consumption. Inhibition of tumor glycolysis elevated available glucose levels in the TME and promoted the ability of CTLA-4 blockade to induce Treg cell fragility associated with IFN-γ production and development of anti-tumor immunity (210). Among several new immunotherapy targets, GITR activation can promote effector T cell function and inhibit Treg cell function. In line, therapeutic application of the agonist anti-GITR monoclonal antibody DTA-1 in B16F10 melanoma-bearing mice induced regression of tumors accompanied by decreased accumulation of intra-tumor Treg cells due both to loss of *Foxp3* expression and impaired infiltration (211). Complete *Foxp3* loss in intra-tumoral Treg cells correlated with a dramatic decrease in *Helios* expression and was associated with the upregulation of Tbet, Eomes and INF-γ. Interestingly, tumor preconditioning and the TME were essential for GITR dependent modulation of *Foxp3* expression since Treg cells not exposed to the TME did not lose *Foxp3* expression following treatment with DTA-1. Therefore, agonist GITR antibodies are promising immunotherapeutic tools since they may abolish the immunosuppressive TME without ensuing the autoimmune side effects (97, 212).

As thoroughly described in the previous paragraphs of this review Treg functional instability results on the one hand in tumor eradication but on the other hand in autoimmune manifestations. Whether and when ICI-induced Treg functional instability participates in the development of irAEs remains unexplored. In line with this notion our group has recently identified a Th-like inflammatory signature in Treg cells isolated from peripheral blood of individuals with diverse cancer types developing irAEs following immunotherapy with anti-PD-1. This intense transcriptional reprogramming of Treg cells was characterized by enhanced enrichment in transcripts such as *Ifng, Stat1, Rorc* and *Stat3*, supporting the notion of a breakdown in mechanisms of self-tolerance in individuals with solid tumors developing irAEs upon ICI immunotherapy (213). Moreover, human Treg cells isolated from individuals with irAEs experience a robust metabolic reprogramming, enriched in signatures associated with mitochondrial dysfunction and oxidative stress-induced cell death (119, 213).

## Conclusions, Challenges, and Open Questions

It is well established that Treg cells play a pivotal role in maintenance of immune homeostasis and also appear to regulate the outcome of diverse pathological situations. At the same time, Treg cells can be characterized by an increased plasticity influenced by several parameters such as the cytokine microenvironment, the strength of antigen recognition, the anatomical site that Treg cells reside etc. Shedding light on the mechanisms that underlie the induction of Treg cell plasticity holds tremendous therapeutic potential in cancer in which Treg suppressive function dominates the tumor immune evasion mechanisms, but also in diseases with aberrancy in Treg cell activity such as autoimmunity and transplantation. A major caveat in performing this task, remains the lack of specific markers to precisely distinguish not only Treg cells from T effectors, but also the different subsets of Treg cells. As we discussed above, Treg cells come in various flavors and adopt a different transcriptional program tailored to the specific microenvironment. Thus, single cell (34) genetic (i.e. RNAseq) and epigenetic (i.e. ATACseq) approaches should provide a comprehensive profiling of Treg cells in each context, which may guide the therapeutic decisions and may reveal unique markers to assist the isolation, functional characterization and targeting of these cells. In addition, particular emphasis should be placed on the metabolic profile of the Treg cells, since over the last decade elegant studies highlight that, metabolic cues determine the functional properties of Treg cells. Therefore, identification of metabolites and pathways that interfere with the Treg cells stability program in a disease setting should be determined.

The fact that Treg cells express major checkpoint inhibitors, which constitute therapeutic targets in both solid tumors and hematologic malignancies with impressive results, proposes that Treg cell manipulation could lead to tumor regression. The goal here should be to induce Treg cell fragility or to interfere with Treg cell suppressive function, preferably in an antigen-specific manner, which will allow the re-start of anti-tumor immunity. To achieve this, we should understand the mechanisms that mediate Treg cell fragility and to identify novel molecules/pathways that could be targeted in order to induce fragile or ex-Treg cells. A major challenge, which still remains is the precise targeting of clonal Treg cells to promote tumor regression without disturbing immune homeostasis. One could hypothesize that ICI give rise to the development of the wide spectrum of irAEs since they also imprint on the peripheral pool of Treg cells impairing their suppressive activity. Although, direct proof is still missing, generated data from our group discussed above, favor the hypothesis and highlight the necessity to unravel the *in vivo* mechanisms of Treg cell-mediated suppression and how immunotherapy interferes with them in specific pathogenic contexts.

Characterization of Treg cell fragile program, may also assist in the development of Treg cell therapies in autoimmunity and transplantation. Various clinical trials and preclinical studies highlight the potential of Treg cell adoptive therapies to treat autoimmune pathologies such as type 1 diabetes, SLE and autoimmune central nervous system disease as well as to induce tolerance during solid organ and bone marrow transplantation. One of the major challenges that have impeded the adoption of Treg cell therapies in the clinic is the lack of knowledge on Treg cell stability in the highly inflammatory environments of the aforementioned pathologic conditions. Considering the advances on the genome editing techniques and the success of engineered chimeric antigen receptor T cell therapies, generation of CAR Treg cell-based therapies has been envisioned, aiming to dampen inflammation and to restore immune tolerance. To this end, the ability to introduce multiple editing events per single cells with the CRISPR technologies, set the stage for generation of Treg cells carrying suicide genes which mediate their fragility, along with genes empowering their function, mediate their trafficking and delivering suppressive mediators. Combined with expression of antigen specific receptors, these engineered Treg cells should hold a tremendous therapeutic potential for inflammatory diseases.

Finally, over the last years the appreciation of the Treg cell residency in non-lymphoid tissues (nltTregs) like skin, adipose tissue, lung and bone marrow, with the ability to control local inflammatory responses and to express diverse transcriptional programs compared to lymphoid tissue Treg cells, have generated new challenges and questions on the Treg cell biology field. In regards to Treg cell stability, it is of interest to be determined whether and how nltTregs respond to local inflammatory and metabolic cues and if this signals imprint on their stability and on *Foxp3* expression. As an example, Treg cells that reside in adipose tissue have been shown to play an important role in controlling adipose tissue inflammation, while their defects are involved in the pathogenesis of obesity-related metabolic disorders. Towards this, inflammatory cytokines and engagement of major metabolic pathways such mTOR/AKT have been shown to drive Treg cell defects in adipose tissues, however the precise mechanisms leading to Treg cell lose and whether this involves induction of fragility and/or ex-Treg development remain to be determined. Overall, addressing such questions may provide novel strategies for combating chronic inflammation and metabolic disorders but also will aid to the design of rational treatments in cancer.

## Author Contributions

AH, AB, MP, IP AV and TA performed the literature searches and contributed to draft versions of the manuscript. AH and PV wrote and revised the final version of the manuscript. All authors contributed to the article and approved the submitted version.

## Funding

This work was supported by the Hellenic Foundation for Research and Innovation (H.F.R.I) and Stavros Niarchos Foundation (S.N.F), grant #1429 to PV. TA is supported by the European Research Council (ERC) under the European Union’s Horizon 2020 research and Innovation program (grant agreement no. 947975) and by the Hellenic Foundation for Research and Innovation (H.F.R.I.) under the “2nd Call for H.F.R.I. Research Projects to support Post-Doctoral Researchers” (Project Number: 166).

## Conflict of Interest

The authors declare that the research was conducted in the absence of any commercial or financial relationships that could be construed as a potential conflict of interest.

## Publisher’s Note

All claims expressed in this article are solely those of the authors and do not necessarily represent those of their affiliated organizations, or those of the publisher, the editors and the reviewers. Any product that may be evaluated in this article, or claim that may be made by its manufacturer, is not guaranteed or endorsed by the publisher.
